# Cryopreserved mesenchymal stem cells regain functional potency following a 24-h acclimation period

**DOI:** 10.1186/s12967-019-2038-5

**Published:** 2019-08-29

**Authors:** Ben Antebi, Amber M. Asher, Luis A. Rodriguez, Robbie K. Moore, Arezoo Mohammadipoor, Leopoldo C. Cancio

**Affiliations:** 1 United States Army Institute of Surgical Research, 3698 Chambers Pass BLDG 3610, JBSA Fort Sam Houston, San Antonio, TX 78234 USA; 2 Oak Ridge Institute for Science and Education, Oak Ridge, TN USA; 3 Biomedical Engineering Department, University of Texas at San Antonio, San Antonio, TX USA

**Keywords:** Cell therapy, Mesenchymal stem cells, Cryopreservation, Freshly thawed, Acclimation

## Abstract

**Background:**

Mesenchymal stem cells (MSCs) are attractive cell-therapy candidates. Despite their popularity and promise, there is no uniform method of preparation of MSCs. Typically, cells are cryopreserved in liquid nitrogen, thawed, and subsequently administered to a patient with little to no information on their function post-thaw. We hypothesized that a short acclimation period post-thaw will facilitate the recovery of MSC’s functional potency.

**Methods:**

Human bone-marrow-derived MSCs were divided into 3 groups: FC (fresh cells; from existing culture); TT (thawed + time; acclimated for 24 h post-thaw); and FT (freshly thawed; thawed and immediately used). The 3 groups were analyzed for their cellular and functional potency.

**Results:**

Phenotypic analysis demonstrated a decrease in CD44 and CD105 surface markers in FT MSCs, with no change in the other two groups. All MSCs were able to differentiate down the osteogenic and chondrogenic lineages. In FT cells, metabolic activity and apoptosis was significantly increased with concomitant decrease in cell proliferation; clonogenic capacity; and key regenerative genes. Following 24-h acclimation, apoptosis was significantly reduced in TT cells with a concomitant upregulation in angiogenic and anti-inflammatory genes. While all MSCs significantly arrested T-cell proliferation, the TT MSCs were significantly more potent. Similarly, although all MSCs maintained their anti-inflammatory properties, IFN-γ secretion was significantly diminished in FT cells.

**Conclusions:**

These data demonstrate that FT MSCs maintain their multipotent differentiation capacity, immunomodulatory function, and anti-inflammatory properties; yet, various aspects of cell characteristics and function are deleteriously affected by cryopreservation. Importantly, a 24-h acclimation period ‘reactivates’ thawed cells to recover their diminished stem-cell function.

## Background

During the last decade, mesenchymal stem cells (MSCs) have emerged as a potent cell-based therapy for a wide spectrum of indications due to their immunomodulatory and anti-inflammatory properties, as well as their capacity to differentiate into cells of mesenchymal origin (osteoblasts, adipocytes, and chondrocytes) [1, 2]. Yet, despite the popularity and promise, there is no uniform method for the preparation and administration of MSCs [1].

Clinical trials involving allogenic MSC therapy typically require 10^7^–10^9^ cells. To generate these large numbers, cells are expanded in vitro and subsequently cryopreserved in liquid nitrogen (LN_2_). The purpose of cryopreservation is to maintain viability by slowing down metabolic processes for long-term storage [3]. To accomplish this, MSCs are typically cryopreserved in 10% dimethyl sulfoxide (DMSO) together with a protein, such as fetal bovine serum (FBS), to sustain cell viability. The DMSO serves as the cryoprotectant to prevent formed ice crystals from rupturing the cell membrane during the slow freezing process (1 °C/min) [4]. Generally, this conventional practice has been confirmed to preserve the viability and differentiation potential of MSCs [5–8]; however, various studies have demonstrated that cryopreservation may impact MSC function post-thaw [9–11]. Despite this knowledge, cryopreservation and preparation of MSCs in clinical studies remain suboptimal. For MSCs to be efficacious in the clinic, effects of cryopreservation on MSC function require further elucidation.

Studies have employed different strategies to optimize the freezing and thawing process for cell-based therapies, albeit with limited success. Since DMSO is cytotoxic and has been shown to induce differentiation and epigenetic modification in stem cells [12, 13], lower concentrations and alternative cryoprotectants have been investigated, such as polyvinylpyrrolidone, glycerol, polyethylene glycol, and trehalose [14, 15]. Other than DMSO substitutes, various cryopreservation parameters have been examined including different cooling protocols, reducing or altogether eliminating animal serum from the cryopreservation solution (e.g., human serum albumin in lieu of FBS), as well as modifying the temperature and periods that are used for cold storage [16, 17]. Nevertheless, regardless of the reagents and methods used, some effects of cryopreservation are expected. Therefore, strategies to mitigate these effects should be developed.

In this study, we compared the characteristics and functional potency of human bone-marrow-derived MSCs before cryopreservation, immediately after thawing, and after 24 h of acclimation post-thaw. The objective of the study was to elucidate the effects of cryopreservation on MSCs with the underlying hypothesis that a short acclimation period can facilitate the recovery of their therapeutic function.

## Materials and methods

### Mesenchymal stem cell culture

Human MSCs were isolated from fresh bone-marrow mononuclear cells purchased from AllCells (Alameda, CA). The MSCs were expanded in complete culture media (CCM) consisting of α-MEM supplemented with 15% heat-inactivated, lot-selected fetal bovine serum (FBS, Atlanta Biologicals, Flowery Branch, GA), 1% antimicrobial/antimitotic, and 1% l-glutamine, as previously described [18]. Following expansion, the MSCs were harvested using 0.25% Trypsin/EDTA, re-suspended in cryopreservation medium, composed of 90% FBS and 10% dimethyl sulfoxide (DMSO), and cryopreserved in − 80 °C overnight and subsequently in liquid nitrogen (LN_2_) for 7 weeks. One week prior to experimentation, MSCs were thawed, expanded in CCM, and harvested on experimentation day to create the ‘fresh cells’ (FC) group. One day prior to experimentation, another group of MSCs were thawed, acclimated for 24 h in standard tissue-culture flasks, and harvested on experimentation day to create the ‘thawed + time’ (TT) group. On the day of experimentation, a third group of MSCs were thawed out of preservation and used immediately to create the ‘freshly thawed’ (FT) group (Table 1). MSCs from the 3 groups were identical in regards to passage number (P3) and population doublings (PDs: 18.3).

**Table 1:** Tabulated description of experimental groups of mesenchymal stem cells

FC	Fresh cells; cells expanded in culture for 7 days prior to experimentation
TT	Thawed + time; cells acclimated for 24 h prior to experimentation
FT	Freshly thawed; cells thawed on day of experimentation; no acclimation time

### Immunophenotyping

Cells were examined by flow cytometry for the expression of common MSC markers. An MSC Analysis Kit (BD Biosciences, San Jose, CA) was used for assessing human MSCs. Cells were stained with pre-conjugated antibodies based on the manufacturer’s instruction. Briefly, MSCs were incubated with staining buffer containing 1% bovine serum albumin and Fc blocker (BioLegend, San Diego, CA) for 10 min at a cell concentration of 1 × 10^6^/ml in order to reduce non-specific binding. Then, the antibody cocktail for MSC-positive markers (CD90-FITC, CD105-PerCP-Cy5.5, CD73-APC), and negative markers (CD45-PE, CD34-PE, CD11b-PE, CD19-PE, HLA-DR-PE) was added to the cells. In addition, in two separate tubes, PE-conjugated mouse monoclonal CD44-PE antibody or PE-conjugated mouse monoclonal CD142-PE antibody (tissue factor, BD, Biosciences, San Jose, CA) was added to MSCs. After 20 min of incubation at 22 °C, cells were washed to remove excess antibodies. Analyses were carried out on a BD FACSCanto II or on a BD FACSCelesta using the BDFACS Diva software, as previously described [19].

### Multipotent differentiation capacity

A multi-differentiation assay was used to evaluate the multipotent capacity of MSCs to give rise to osteoblasts and chondrocytes using a commercially-available differentiation media (StemPro Differentiation Kits, Thermo Fisher Scientific), as previously described [20]. For this purpose, the MSCs from the different groups were cultured in 8-well chamber slides for histological evaluation. Briefly, for osteogenic differentiation, cells were cultured in osteogenic differentiation media. The differentiation media were replaced twice weekly. After 21 days, differentiation was assessed by quantifying calcium deposits using alizarin red staining. For chondrogenic differentiation, 5-μl droplets of cell solution at a density of 1.6 × 10^7^ cells/ml was seeded in the center of a multi-well plate to form a micromass cultured for 2 h, and then induced using the chondrogenic differentiation media. Differentiation media were changed every other day. After 14 days, chondrogenic differentiation was assessed by Alcian Blue staining of sulfated proteoglycans.

### Cell apoptosis

Cells apoptosis was measured by flow cytometry using Annexin V kit (BioRad, Hercules, CA) per the manufacturer’s instructions. Briefly, MSCs were collected and washed in PBS containing 1% bovine serum albumin (BSA). Then, MSCs were resuspended in 1× annexin binding buffer at a cell concentration of 1.5 × 10^6^/ml and subsequently incubated for 10 min with annexin V-FITC in the dark. Propidium iodide (PI) was added to MSCs to be immediately analyzed by FACSCelesta (BD Biosciences, San Jose, CA) using the BDFACS Diva and FLowJo software. Cell fragments were removed by morphological gating. Cells negative for annexin V-FITC and PI were considered viable; annexin V-FITC positive and PI negative were considered early apoptotic; and annexin V-FITC positive and PI positive were considered late apoptotic/necrotic.

For qualitative assessment of proliferation, MSCs were stained with a fluorescent Live/Dead Cell Viability Kit, according to the manufacturer’s instruction (Life Technologies, Grand Island, NY) and as previously described [18]. In this live/dead assay, the cytoplasm of viable cells is stained green (Ex/Em 495 nm/515 nm) and the nucleus of dead cells is stained orange (ex/em 528 nm/617 nm).

### Cell metabolic and proliferation activity

MSCs were evaluated for their metabolic activity using the Vybrant assay (Thermo Fisher Scientific, Waltham, MA) according to the manufacturer’s instructions. In this assay, non-fluorescent resazurin (R-12204) is reduced by viable cells to red-fluorescent resorufin. To perform this, MSCs were seeded at 1000 cells/cm in triplicates and their media evaluated along three different time points on days 3, 7, and 10. At each time point, the fluorescent product was measured at wavelengths of 563/587 nm using a SpectraMax i3X system (Molecular Probes, Eugene, OR).

Following the metabolic assay, the MSCs were placed in a cell-lysis buffer (Cell Signaling Technology, Danvers, Massachusetts). Following lysis, the multi-well plates were stored at − 80 °C until batch analysis. Next, plates were thawed and DNA concentration was measured using the Quant-iT PicoGreen assay (Invitrogen, Carlsbad, CA) to evaluate cell proliferation, as previously described [21]. Briefly, an ultrasensitive fluorescent nucleic acid stain was used to quantify double-stranded DNA in solution. Samples were prepared by diluting with 1× TE buffer (1:100) then plated in duplicates. PicoGreen working solution was then added to pre-diluted samples. Plates were run on a SpectraMax i3X system (Molecular Probes, Eugene, OR) and fluorescence measured at a wavelength of 502/523 nm.

### Colony-forming unit fibroblast (CFU-F) assay

The colony-forming unit fibroblast (CFU-F) assay was used as an indicator of progenitor cells, as previously described [21]. Briefly, MSCs were plated at 100 and 200 cells per well on 6-well plates in a total of 3 ml of CCM per well. Media were changed every 3–4 days and the cells were allowed to grow for 7–10 days. Prior to the overlap of colonies, cells were washed with PBS and fixed with chilled methanol for 10 min at room temperature. Next, the plates were allowed to air dry and stained with Giemsa to allow for visualization. Colonies larger than 50 cells were enumerated and reported as CFUs/well.

### Gene expression

To determine gene expression via quantitative real-time polymerase chain reaction (qRT-PCR), total RNA was extracted from MSCs using Trizol (Thermo Fisher Scientific) and reverse-transcribed using a High Capacity cDNA Archive Kit (Applied Biosystems, Foster City, CA). The transcripts of interest were amplified from cDNA using Taqman Universal PCR Master Mix and all the primers were purchased from Applied Biosystems. Amplification and detection were carried out with a StepOnePlus Real-Time PCR System (Applied Biosystems) for the pertinent genes (Table 2). For normalization, the MSC FC group was used as the reference sample. 18s was used as the housekeeping gene. Gene expression is expressed as a relative quotient (RQ) calculated from ΔΔCt of the sample of interest, where C_T_ is the threshold cycle.

$$\Delta C_{T\;Gene\;of\;Interest} = C_{T\;Housekeeping } - C_{T\;Gene\; of\;Interest}$$

$$\Delta \Delta C_{T\;Gene\;of\;Interest} = \Delta C_{T\;Gene\;of\;Interest} - \Delta C_{{\begin{array}{*{20}c} {T\;Reference} \\ {} \\ \end{array} }}$$

$$RQ = 2^{{ - \Delta \Delta C_{T\;Gene\;of\;Interest} }}$$

**Table 2:** Genes analyzed by qRT-PCR

HMGB1	High mobility group box 1; DNA binding protein involved in tissue damage
TRL4	Toll-like receptor 4; play role in activation of innate immunity
TNF-α	Tumor necrosis factor alpha; upregulated in inflammation
CYCS	Cytochrome c; involved in initiation of apoptosis
BCL2	B-cell lymphoma 2; regulates apoptosis
BAX	BCL-2-like protein 4; apoptosis activator
CASP3	Caspase 3; apoptosis activator
NANOG	Transcription factor essential for pluripotency
SOX2	Sex determining region Y-box 2; transcription factor essential for pluripotency
OCT-4	Octamer-binding transcription factor 4; transcription factor essential for pluripotency
TNFAIP6	Tumor necrosis factor-inducible gene 6 protein; involved in macrophage polarization (M1 to M2 phenotype)
VEGF	Vascular endothelial growth factor; essential for angiogenesis
HMOX-1	Heme oxygenase 1; anti-inflammatory upregulates IL-10 and IL-1ra
STC-1	Stanniocalcin 1; regulation of metabolism and calcium/phosphate homeostasis
CAT	Catalase; catalyzes hydrogen peroxide to protect cell from reactive oxygen species
VCAM1	Vascular cell adhesion molecule 1; mediates adhesion of various immune cells
ICAM1	Intercellular adhesion molecule 1; mediates adhesion of leukocytes when activated

### Mixed lymphocyte reaction (MLR) assay

MSCs were plated at 5 × 10^4^ cells per well, in triplicates, in a 24-well plate. To allow for cell attachment, MSCs were incubated for at least 2 h prior to addition of mononuclear cells (MNCs). Human MNCs were isolated from peripheral blood of consenting donors using Ficoll-Paque using an institutional IRB-approved protocol. Carboxyfluorescein succinimidyl ester (CFSE; Sigma Aldrich, St. Louis, MO) was used to fluorescently label the MNCs at a concentration of 5 µM/ml. The MNCs were mixed well and incubated for 5 min in the dark. After incubation, the MNCs were washed twice and resuspended in MNC media (RPMI 1640 medium supplemented with 10% FBS, 1% Anti–Anti, and 1% l-glutamine) at a concentration of 0.5 × 10^6^ cells/ml. Then, the MNCs were stimulated with Phytohaemagglutinin (PHA) at a concentration of 5 µg/ml. Next, 1 ml of stained, stimulated MNCs was added to MSCs and incubated for 96 h. In addition, MNCs were plated in the absence of MSCs as a positive control. After 96 h, the MNCs and conditioned media (CM) were collected for further analysis. The collected MNCs were centrifuged, and resuspended in filtered PBS containing 1% BSA and Fc blocker (BioLegend, San Diego, CA). MNCs were incubated for 10 min in dark and subsequently labeled with pre-conjugated CD3-APC antibody (BD Pharminogen) for another 15 min. After washing, 7AAD viability dye (BD Pharminogen) was added and the cells incubated for 5 min. Proliferation rate of the live CD3-positive T cells was analyzed by FACSCelesta or FACSCanto II (BD Biosciences, San Jose, CA) using the BDFACS Diva and FlowJo software. The CM were analyzed with a Milliplex kit for the following cytokine/chemokines: IL-1α, IL-1β, IL-1ra, IL-4, IL-6, IL-8, IL-10, IL-12, MCP-1, and IFN-γ. Protein content was normalized to total protein using the Pierce™ 660-nm protein assay (Thermo Scientific) according to the manufacturer’s instructions.

### Anti-inflammatory assay

MSCs were seeded at 5 × 10^4^ cells per well, in quadruplicates, in a 24-well plate and placed in standard culture conditions for 2 h to allow for cell attachment. MNCs were prepared at a concentration of 0.5 × 10^6^ cells/ml in MNC media. Prior to addition of lipopolysaccharide (LPS), MNC control cells were set aside. Next, LPS was added to the MNCs at a concentration of 50 ng/ml and 0.5 × 10^6^ of the stimulated MNCs were added to the MSCs. Following 18 h incubation, the co-cultures supernatant was collected, cell debris was removed via centrifugation, and the resulting conditioned media (CM) were analyzed for levels of IFN-γ, TNF-α, IL-1RA, IL-1β, and IL-10 using a Milliplex kit (Millipore, Billerica, MA). Protein content was normalized to total protein using the Pierce™ 660-nm protein assay (Thermo Scientific) according to the manufacturer’s instructions.

### Statistical analysis

Results are presented as means ± standard errors of the mean. All statistical tests were performed with the aid of GraphPad Prism version 7.01. Experimental data were analyzed with a one-way analysis of variance (ANOVA) followed by Tukey’s Multiple Comparisons post-test. Evaluation and exclusion of outliers was performed using the ROUT method; a probability (*p*-) value less than 0.05 was considered statistically significant.

## Results

### Immunophenotype and multipotent differentiation

**Fig. 1:**
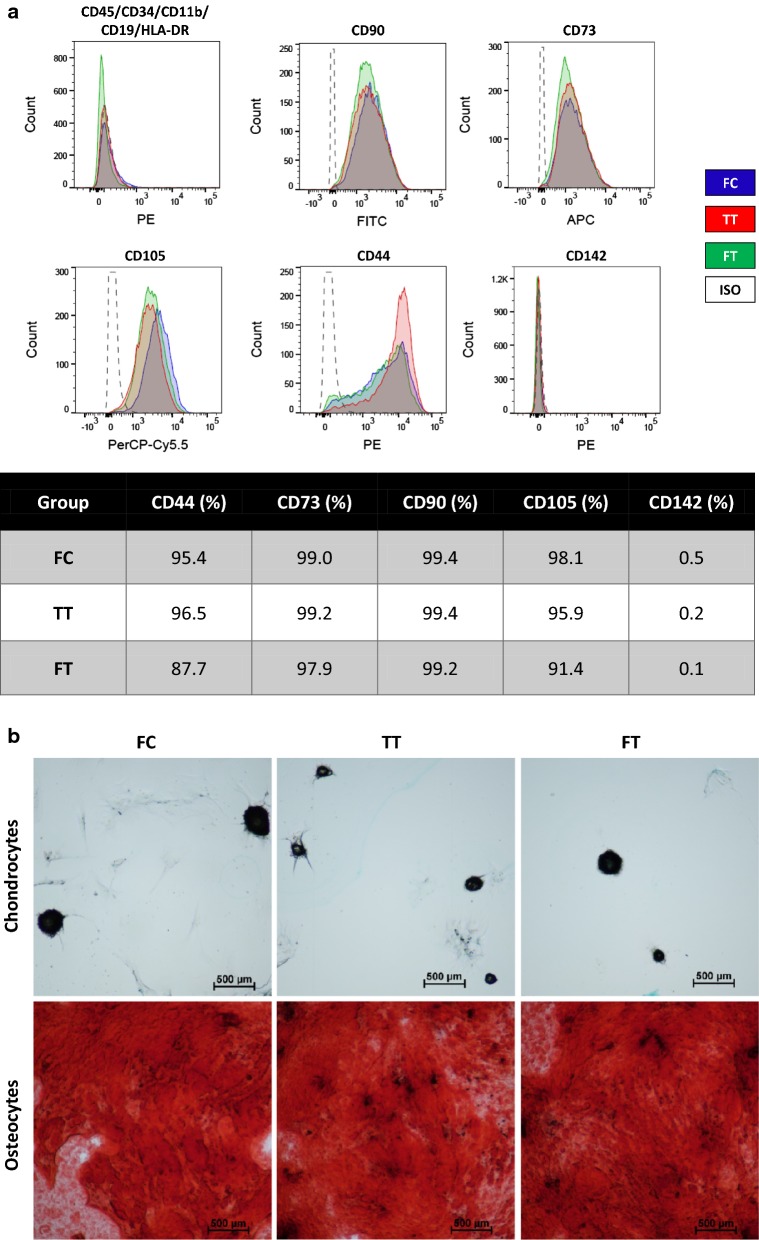
Immunophenotype and multipotent capacity of MSCs from the 3 different groups. **a** Flow cytometry analysis revealed a decrease in CD44 and CD105 surface markers in FT cells whereas the other two groups maintained surface expression greater than 95%. **b** MSCs from all groups were able to differentiate down the osteogenic and chondrogenic lineages. Images were acquired using a 4x objective

To elucidate whether cryopreservation had deleterious effects on MSC phenotype, differentiation capabilities and surface expression of common MSC markers were evaluated. Flow cytometry analysis revealed reduced expression of CD44 (88%) and CD105 (91%) surface markers in the freshly thawed (FT) cells, whereas the other two groups maintained surface expression over 95%. Expression of CD73 and CD90 remained unchanged and above 95% among the 3 groups. Importantly, no tissue factor (CD142, indicative of pro-thrombogenic disposition) expression was detected in any of the MSC groups (Fig. 1a). Moreover, MSCs from all groups were able to differentiate down the osteogenic and chondrogenic lineages with no apparent differences among the 3 groups (Fig. 1b).

### Cell metabolism, proliferation, clonogenicity, and apoptosis

**Fig. 2:**
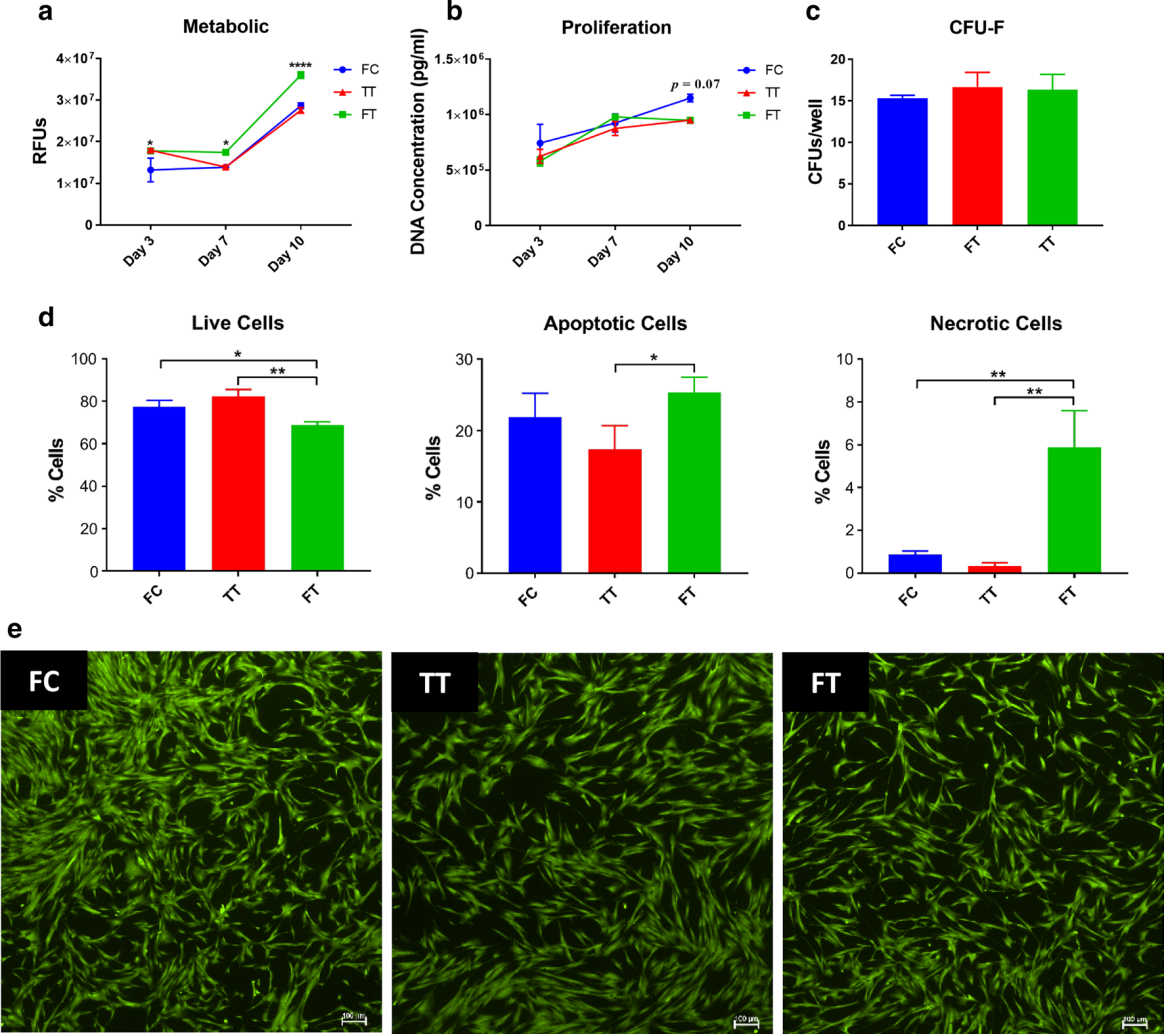
Functional characteristics of MSCs from the 3 different groups. **a** The metabolic activity was significantly higher in FT MSCs at all time points. **b** The proliferation of FC cells was higher at day 10 compared to TT and FT cells (*p* = 0.07). **c** The clonogenic capacity of FC MSCs was significantly higher compared to FT cells. **d** Apoptosis was significantly reduced in TT MSCs following a 24-h acclimation period. **e** Live cells growing in culture from the 3 groups exhibiting similar morphology; scale bars are 100 microns in length. *p < 0.05; ****p < 0.0001

The effect of cryopreservation on MSC function was also investigated. On day 3, the metabolic activity was significantly higher (*p* < 0.05) in FT and acclimated (TT) cells as compared to fresh cultured (FC) cells. On days 7 and 10, the metabolic activity of FT cells was significantly higher (*p *< 0.05 and *p* < 0.0001, respectively) compared to the other two groups (Fig. 2a). Proliferation of FC cells was higher, albeit not significantly, at day 10 compared to TT and FT cells (*p* < 0.07) (Fig. 2b). Similarly, the clonogenic capacity of FC MSCs was significantly higher (*p* < 0.05) compared to FT cells (Fig. 2c). Since standard cryopreservation and thawing techniques can affect viability, cell apoptosis was examined via flow cytometry. Percent live cells was significantly lower in FT MSCs compared to the FC (*p* < 0.05) and TT (*p* < 0.01) groups. Correspondingly, percent apoptotic and necrotic cells was significantly higher in FT MSCs, as compared to the other two groups (Fig. 2d). Fluorescent images demonstrated similar cell morphology and overall viability among the different groups (Fig. 2e).

### Gene expression

**Fig. 3:**
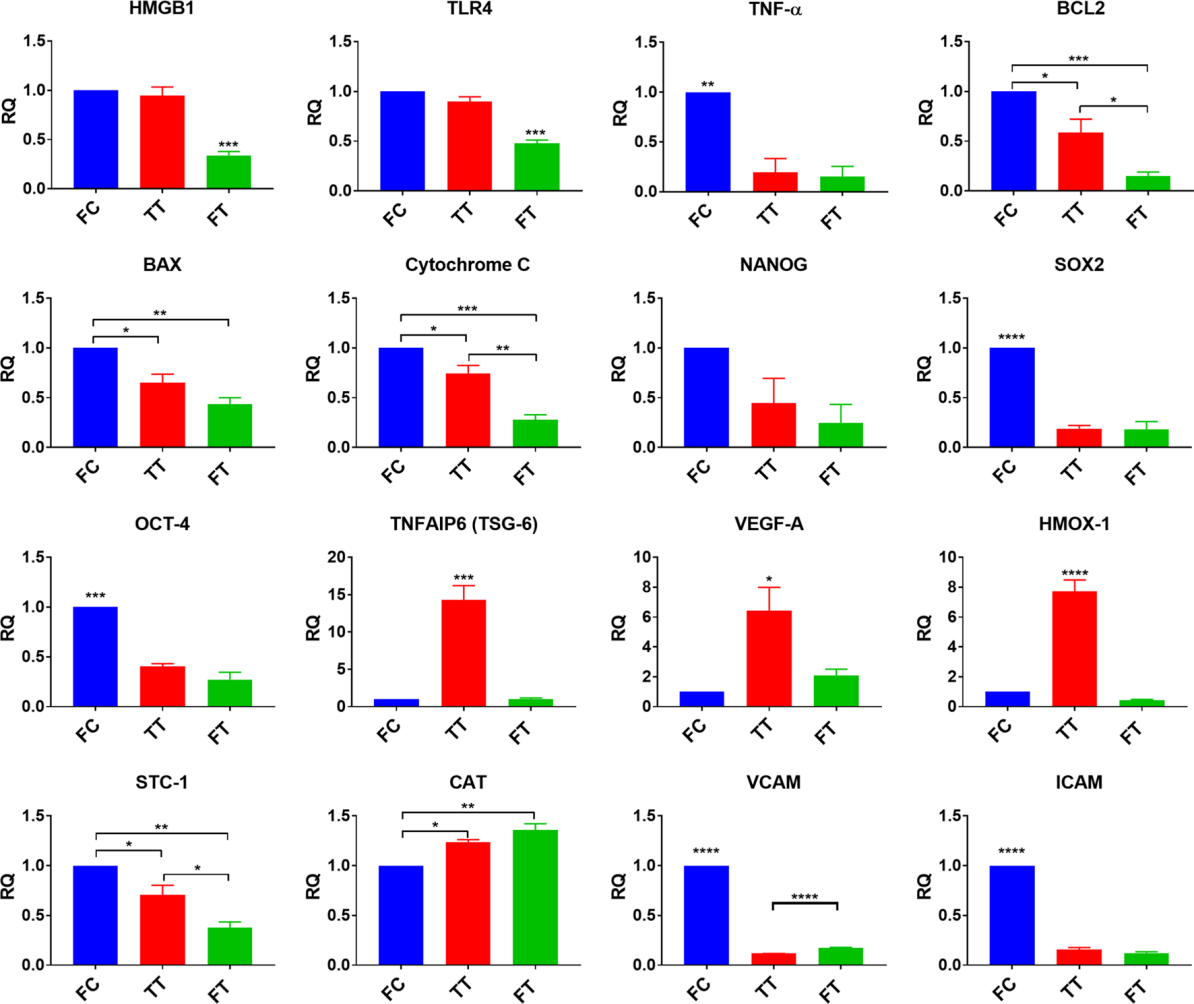
Gene expression of MSCs from the 3 different groups. Gene expression analysis revealed a downregulation in the tissue injury-related genes, HMGB1 and TLR-4 in FT MSCs. Apoptosis-related genes (cytochrome C, BAX, BCL-2) and the pro-inflammatory TNF-α were downregulated in both TT and FT MSCs. The anti-inflammatory/anti-oxidant genes HMOX and TSG-6 as well as the angiogenesis gene VEGF were upregulated in TT cells. Finally, the stem cell genes, OCT-4, NANOG, and SOX2 as well as the anti-inflammatory gene, STC-1 were downregulated in both TT and FT cells. *p < 0.05; **p < 0.01; ***p < 0.001; ****p < 0.0001

MSC response to cryopreservation and thawing was evaluated for genes involved in tissue injury, apoptosis, pluripotency, angiogenesis, and anti-inflammatory function. Gene expression analysis revealed a downregulation in the tissue-injury-related gene, HMGB1, and its receptor, TLR-4, in FT MSCs compared to the other two groups (*p* < 0.001). The apoptosis-related genes, BCL-2, BAX, and cytochrome C were significantly downregulated in TT (*p* < 0.05) and FT (*p* < 0.01) cells as compared to FC cells. The stem-cell genes SOX2 (*p* < 0.0001) and OCT-4 (*p* < 0.001) were significantly downregulated with concomitant upregulation in the anti-oxidant gene, catalase, in both TT and FT cells, compared to FC cells. The adhesion genes VCAM1 and ICAM1 were significantly downregulated in both TT and FT cells (*p* < 0.0001) with a more significant downregulation in VCAM1 in TT cells (*p* < 0.0001), as compared to the other two groups. Significant upregulation in the anti-inflammatory genes TSG-6 (*p* < 0.001) and HMOX-1 (*p* < 0.0001), as well as the angiogenesis gene VEGF (*p* < 0.05) was seen in TT MSCs compared to the other two groups. The pro-inflammatory TNF-α gene was significantly downregulated (*p* < 0.01) in both TT and FT MSCs (Fig. 3).

### Immunosuppressive and anti-inflammatory function

**Fig. 4:**
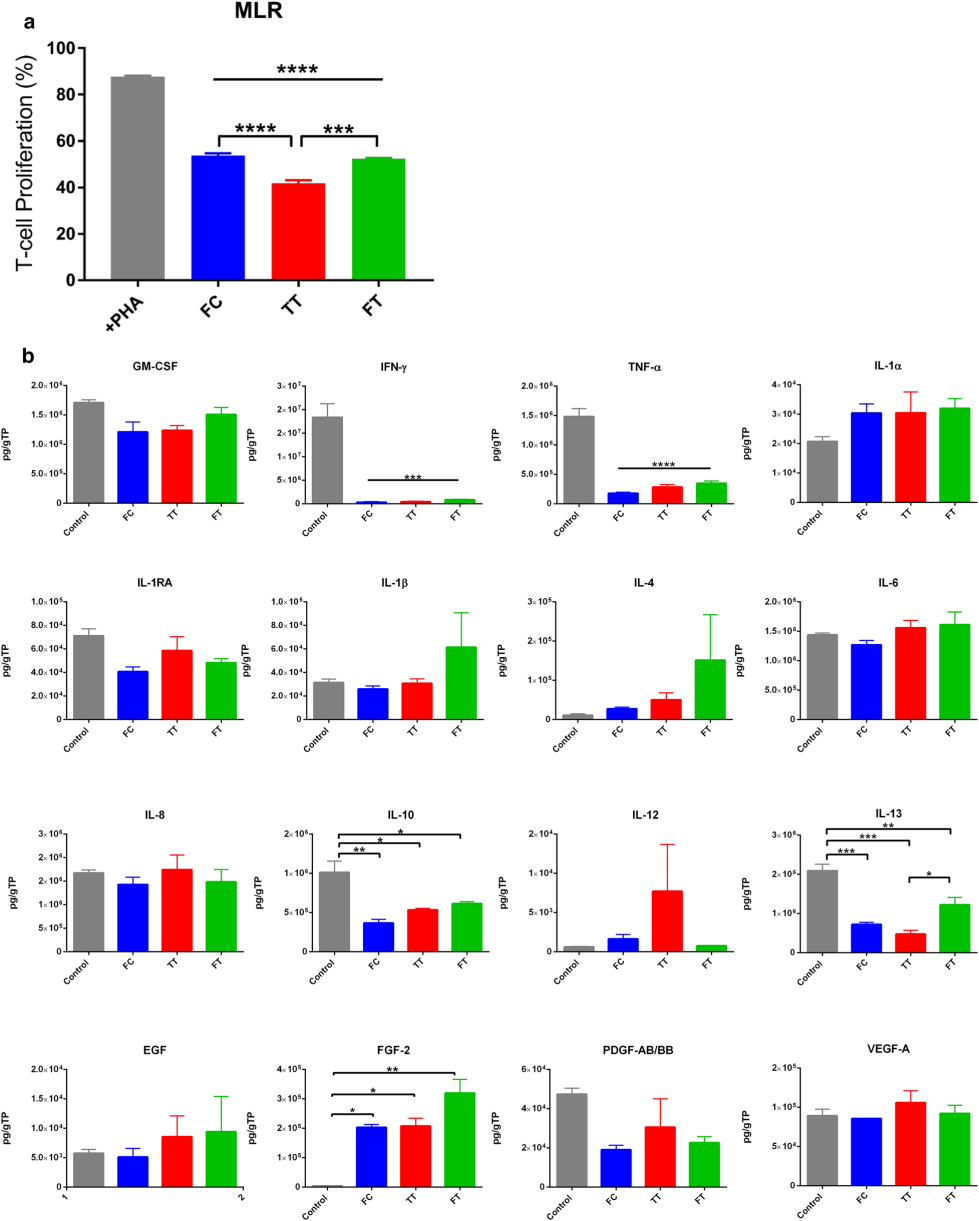

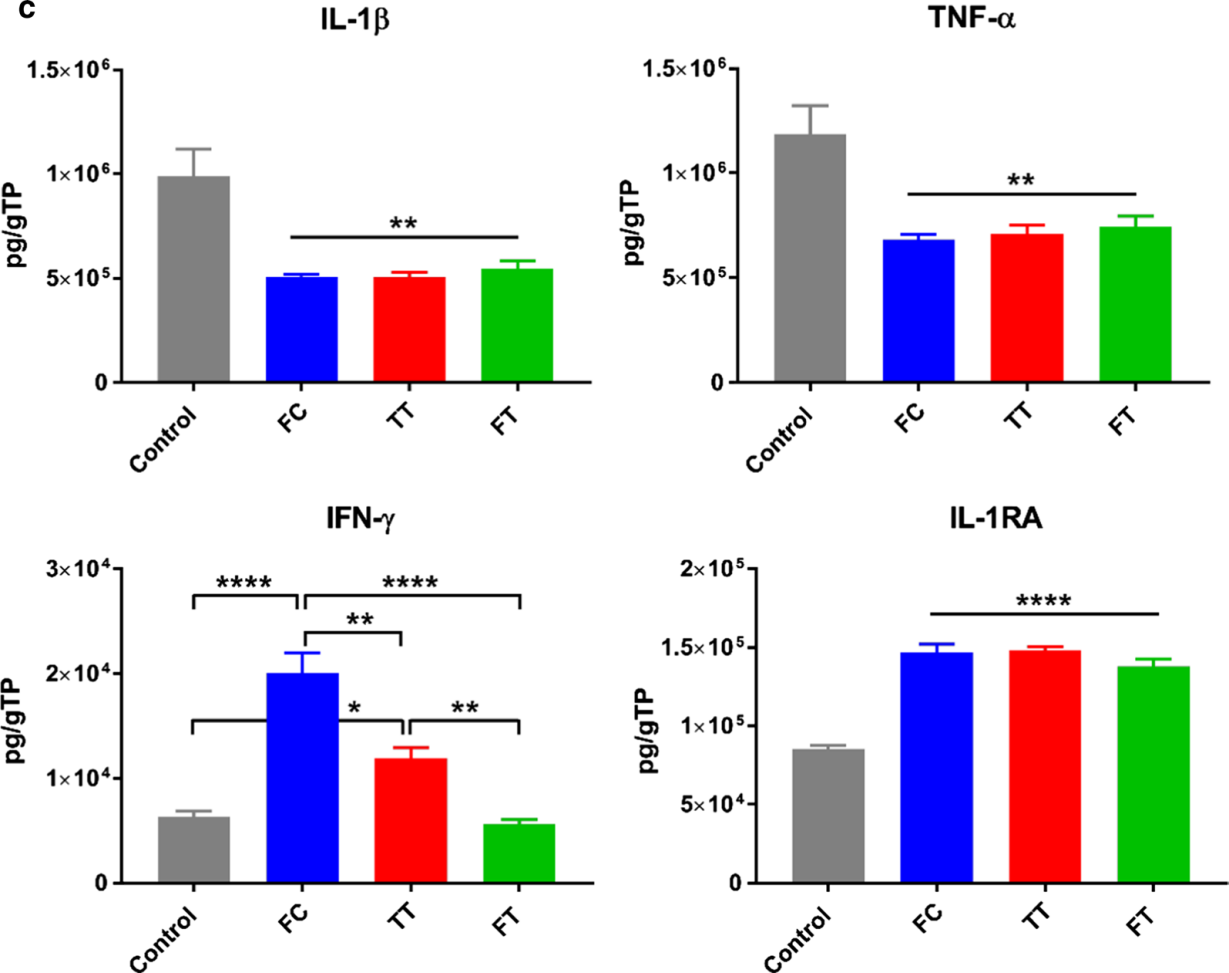
Immunosuppressive and anti-inflammatory properties of MSCs from the 3 groups in a co-culture system. **a** All groups were able to suppress T-cell proliferation, but the TT group performed significantly better, whereas the FT cells were the least potent. **b** All MSCs were able to suppress the secretion of the pro-inflammatory cytokines IFN-γ, TNF-α, and IL-13. **c** Following a LPS challenge, all MSCs were able to suppress the secretion of the pro-inflammatory cytokines IL-β and TNF-α with concomitant increase in the secretion of the anti-inflammatories IL-1RA and IL-10. Additionally, the FC cells secreted significantly more IFN-γ than all other groups. *p < 0.05; **p < 0.01; ***p < 0.001; ****p < 0.0001

Immunosuppression was also examined among the 3 groups of MSCs using a MLR assay. All groups were able to significantly suppress T-cell proliferation (p < 0.001) as compared to controls; however, the effect of the TT MSCs was significantly more potent (*p* < 0.0001) whereas the FT cells were significantly less potent (Fig. 4a). Immunomodulation of MSCs was also examined by measuring their secreted factors in the MLR co-culture system. MSCs from all groups significantly suppressed the secretion of the pro-inflammatory mediators, IFN-γ (*p* < 0.001), TNF-α (*p* < 0.0001), and IL-13 (*p* < 0.001 for FC and TT; *p* < 0.01 for FT), with concomitant increase in FGF-2 (*p* < 0.05 for FC and TT; *p* < 0.01 for FT), compared to controls (Fig. 4b).

The anti-inflammatory function of the different MSCs was evaluated by measuring the secretion of pro/anti-inflammatory mediators in a co-culture system. In response to a LPS challenge, all MSCs were able to similarly significantly suppress the secretion of IL-1β (*p* < 0.01) and TNF-α (*p* < 0.01) while promoting the secretion of the anti-inflammatory, IL-1RA (*p* < 0.0001), and IL-10 (over-saturated; data not shown), as compared to controls. However, levels of IFN-γ were significantly higher in FC and TT MSCs compared to controls (*p* < 0.0001 and *p* < 0.05, respectively) and FT cells (*p* < 0.0001 and *p* < 0.01, respectively) (Fig. 4c).

## Discussion

To provide the needed doses for clinical studies, MSCs are expanded in vitro and subsequently frozen for storage/banking purposes [22]. Marquez-Curtis and colleagues reviewed how aspects of cryopreservation, including the type and concentration of cryoprotectants used, cooling rate, storage period, and storage temperature affect the characteristics of MSCs from different tissues. Overall, their findings demonstrate that MSCs retain their biological properties post-thaw despite the rate of cooling and cryoprotectant concentration, but some alteration in cell function is evident [16]. Although cryopreserved cells are typically used in the clinic, recent studies have demonstrated their lower therapeutic efficacy compared to fresh cells [23–26]. Therefore, a short recovery period post-thaw in culture may facilitate a regain of function. In this study, we evaluated 3 groups of MSCs: fresh cells (FC); cells recovered for 24 h post-thaw (TT); and freshly thawed cells (FT).

In line with other studies, our results demonstrate that cryopreservation deleteriously affect cell viability and function [27–30]. Specifically, surface-marker expression of common MSC markers (CD44 and CD105) was diminished in FT cells, whereas surface expression of positive markers remained above 95% in the TT and FC groups, as recommended by the International Society for Cellular Therapy (ISCT) for defining MSCs. In addition, in agreement with other studies [5–7, 31], differentiation was not affected by the cryopreservation process (Fig. 1).

Cell metabolism and proliferation were measured for 10 days in the different MSC groups. On all days, metabolism of FT MSCs was significantly higher (Fig. 2a). Killer et al. reported decreased metabolism of freshly-thawed MSCs immediately post-thaw. They attributed this phenomenon to DMSO and demonstrated that the metabolic activity of MSCs increased on subsequent days post-thaw, which is in agreement with our data [32]. As with the study by Kaplan et al., cryopreservation did not significantly affect cell proliferation, but trends demonstrated higher rates in FC MSCs on day 10 (*p* = 0.07, Fig. 2b) [33]. Similarly, the clonogenic capacity was also higher in fresh MSCs as compared to FT MSCs (*p* < 0.05, Fig. 2c).

Cell viability is expected to be compromised to some extent due to cryopreservation. Previous studies have reported viability rates as low as 50% [23], while others demonstrated MSC viability as high as 90% post-thawing [34]. Viability will depend on the cryopreservation/thawing method, duration in cold storage, and reagents used. In our study, viability of FT MSCs was 69%, which was significantly lower compared to the TT (82%) and FC (77%) groups (*p* < 0.01 and *p* < 0.05, respectively). We also demonstrate that FT cells contained significantly more apoptotic (*p* < 0.05) and necrotic (*p* < 0.01) cells than TT and FC cells (Fig. 2d). This fact should be emphasized since standard viability assessment via trypan blue exclusion merely detects dead cells and does not account for early apoptotic cells. Hence, viability of freshly thawed cells is most likely often overestimated in clinical practice [23]. For example, a recent phase 2a trial completed by Matthay and colleagues reported significant variation in viability (36–85%) post-thaw in MSCs for the treatment of acute respiratory distress syndrome (ARDS). In their study, they postulate that the lack of clinical efficacy post MSC infusion was, in part, likely due to the low viability post-thaw [35].

Evaluation of gene expression demonstrated significant changes due to cryopreservation (Fig. 3). The stem cell genes NANOG, SOX2 and OCT-4, VCAM1 and ICAM1, and the pro-inflammatory gene TNF-α were significantly downregulated following cryopreservation. VCAM1 was significantly downregulated in TT cells compared to both the FC and FT cells. This reduced adhesion potential can prove useful for inflammatory indications, such as ARDS, in which ‘cytokine storm’ activate these proteins excessively and contribute to the disease sequalae. Other than catalase, which protects cells from reactive oxygen species, all genes were significantly downregulated in FT cells. A 24-h acclimation period induced upregulation of various key genes (i.e., HMGB1, TLR-4, BCL-2, BAX, CYCs, and STC-1) to levels significantly higher than FT cells. Interestingly, significant upregulation of TSG-6, VEGF-A, and HMOX-1 genes was seen in TT MSCs as compared to the other two groups. This indicates that a short acclimation period appears to potentiate the therapeutic function of MSCs.

The premise that a short acclimation period potentiates MSC function is also supported by the MLR assay, which informs on the immunosuppressive capacity of MSCs to arrest T-cell proliferation in vitro. We observed that irrespective of the cryopreservation process, all groups were able to significantly suppress T-cell proliferation (*p* < 0.001). However, the TT MSCs were significantly more potent than the other two groups, whereas the FT cells were significantly less potent (Fig. 4a). Whether cryopreservation negatively impacts the immunosuppressive capacity of MSCs remains inconclusive in the literature. Francois and colleagues reported an impaired immunosuppressive capacity following cryopreservation [23]. Likewise, Moll et al. showed a slight, yet significant, reduction in the ability of freshly thawed MSCs to inhibit T-cell proliferation when stimulated with PHA, whereas this difference was not evident in allo-stimulated co-cultures [36]. In contrast, Luetzkendorf et al. demonstrated that cryopreservation had no effect on the ability of MSCs to suppress mitogen-stimulated T-cell proliferation [37]. Unequivocally, fundamental differences in MSC donors, isolation, preparation, expansion, and cryopreservation will dictate cell fate and the degree of clinical response [23, 25, 37]. In this study, we demonstrate that cryopreservation does compromise the immunosuppressive capacity of MSCs, but that freshly thawed cells are still functionally active. Importantly, a short acclimation period restores that functionality to levels even higher than fresh cells in culture.

We also examined the secreted bioactive factors in the conditioned media from the MLR assay. In accordance with their immunosuppressive capacity, MSCs from all groups were able to significantly inhibit the secretion of the pro-inflammatory mediators IFN-γ (*p* < 0.001) and TNF-α (*p* < 0.0001) in a comparable manner. Similarly, levels of the pro-inflammatory IL-13 were suppressed by all MSCs; yet, the TT MSCs were significantly more potent than the FT cells (*p* < 0.05), which indicates that paracrine secretion may be affected by the cryopreservation process (Fig. 4b).

Finally, we assessed the anti-inflammatory capacity of the different MSCs following a LPS challenge. LPS, an endotoxin secreted by Gram-negative bacteria, is commonly used to promote the secretion of pro-inflammatory cytokines to mimic infection in vitro or induce ARDS in vivo. In this study, the secretion of the pro-inflammatory cytokines IL-1β and TNF-α were significantly inhibited in all MSC-treated cultures, irrespective of the cryopreservation process (*p* < 0.01). Likewise, the secretion of the anti-inflammatory cytokines IL-1RA and IL-10 were significantly increased with no differences among the three MSC groups (*p* < 0.0001). In contrast, levels of the pro-inflammatory IFN-γ were significantly elevated in both FC and TT MSCs, as compared to both control (MNCs only) and FT cells. This indicates that following cryopreservation the capacity to secrete IFN-γ is diminished in response to a LPS stimulus, but rebounds (albeit not completely, p < 0.01) after acclimation to levels secreted by fresh cells. It can be proposed that the mechanism responsible for the restoration of immunomodulatory function seen in the TT MSCs is facilitated, in part, via the recovery of IFN-γ levels, as suggested in published literature [23, 26, 36].

To the authors’ knowledge, this study is the first to compare, and report on, cell characteristics and function among 3 groups of MSCs, namely, FC, TT, and FT. In addition, herein, we provide evidence that a short acclimation period, in vitro, provides a temporal window for recovery of cell function following cryopreservation, a method that can be potentially translated to the clinic. In this study, a 24-h time period was selected because it is short enough to be clinically feasible while allowing the cells the needed time for recovery. Yet, it is conceivable that a shorter acclimation time, in the order of hours, may yield the same favorable results. This, however, remains to be elucidated.

This study has some limitations that should be acknowledged. One caveat is the fact that the MSCs were obtained from a single donor. Donor-dependent variation is a known issue in the field of cellular therapies, and, therefore, it is important to verify these results in MSCs from multiple donors, as well as, ideally, in MSCs from various tissue sources (e.g., adipose, umbilical cord, etc.). Another limitation is the lack of in vivo data as these results must be validated in an appropriate animal model prior to clinical translation. Finally, the potential implication of a short in vitro acclimation may complicate the cellular handling process in the clinic, which also introduces the possibility for contamination. It can be proposed, therefore, that this acclimation period can be feasible in vivo. For example, intramuscular administration of MSCs is gaining favor as it provides MSCs with sustained dwelling time in the muscle (as opposed to being phagocytized following systemic administration), as recent evidence suggests. This extended dwelling time may permit the recovery of cell function post-thaw in vivo as they impart their effects via paracrine signaling.

## Conclusion

We show that cryopreservation indeed compromises various aspects of cell function. Yet, this loss of potency may be recovered using a short acclimation period. Methods for increased cell viability and function post-thaw are vital for the success of cell therapy in the clinic. We propose that this technique may be employed clinically, especially in light of emerging research showing suboptimal results using cryopreserved cells [35]. If cryopreservation is indeed suboptimal, alternative methods for cell preparation and storage should be developed. For example, freeze-drying has been successfully used to preserve whole platelets for hemorrhage control [38] so it is not unreasonable that the same lyophilization approach can be applied to MSCs [39]. Finally, owing to MSCs’ robust paracrine function, MSC-based products, such as extracellular vesicles (EVs), could emerge as potent alternatives to whole cell therapy. While it is imperative to optimize cell-preparation techniques, logistical challenges must also be considered. A key advantage of EVs include the potential for freeze-drying for long-term storage and delivery to patients in the clinic as well as warfighters in austere environments [40].

## Data Availability

The dataset of the study is available from the corresponding author upon a reasonable request.

## Abbreviations

ANOVA: analysis of variance
ARDS: acute respiratory distress syndrome
BSA: bovine serum albumin
CCM: complete culture medium
CD: cluster of differentiation
CFSE: carboxyfluorescein succinimidyl ester
CFU-F: colony-forming unit fibroblast
CM: conditioned media
Ct: threshold cycle
DMSO: dimethyl sulfoxide
EV: extracellular vesicles
FBS: fetal bovine serum
FC: fresh culture
FT: freshly thawed
GM-CSF: granulocyte-macrophage colony stimulating factor
HMGB1: high-mobility group box 1
ICAM: intracellular cell adhesion molecule
IFN: interferon
IL: interleukin
IL-1RA: IL-1 receptor antagonist
ISCT: International Society for Cellular Therapy
LPS: lipopolysaccharide
MLR: mixed lymphocyte reaction
MNC: mononuclear cell
MSC: mesenchymal stem cell
PD: population doubling
PHA: phytohaemagglutinin
qRT-PCR: quantitative real-time polymerase chain reaction
SOX-2: sex determining region Y-box 2
TLR: toll-like receptor
TNF: tumor necrosis factor
TT: time to thaw
VCAM: vascular endothelial adhesion molecule
VEGF: vascular endothelial growth factor

## References

[1] Antebi B, Mohammadipoor A, Batchinsky AI, Cancio LC (2018). The promise of mesenchymal stem cell therapy for acute respiratory distress syndrome. J Trauma Acute Care Surg.

[2] Antebi B, Pelled G, Gazit D (2014). Stem cell therapy for osteoporosis. Curr Osteoporos Rep.

[3] McLellan MR, Day JG (1995). Cryopreservation and freeze-drying protocols. Introduction. Methods Mol Biol.

[4] Thirumala S, Goebel WS, Woods EJ (2009). Clinical grade adult stem cell banking. Organogenesis.

[5] Liu G, Zhou H, Li Y, Li G, Cui L, Liu W, Cao Y (2008). Evaluation of the viability and osteogenic differentiation of cryopreserved human adipose-derived stem cells. Cryobiology.

[6] Woods EJ, Perry BC, Hockema JJ, Larson L, Zhou D, Goebel WS (2009). Optimized cryopreservation method for human dental pulp-derived stem cells and their tissues of origin for banking and clinical use. Cryobiology.

[7] Bruder SP, Jaiswal N, Haynesworth SE (1997). Growth kinetics, self-renewal, and the osteogenic potential of purified human mesenchymal stem cells during extensive subcultivation and following cryopreservation. J Cell Biochem.

[8] Rust PA, Tingerides C, Cannon SR, Briggs TW, Blunn GW (2006). Characterisation of cryopreserved cells freshly isolated from human bone marrow. Cryo Lett.

[9] Xu X, Liu Y, Cui ZF (2014). Effects of cryopreservation on human mesenchymal stem cells attached to different substrates. J Tissue Eng Regen Med.

[10] Xu X, Liu Y, Cui Z, Wei Y, Zhang L (2012). Effects of osmotic and cold shock on adherent human mesenchymal stem cells during cryopreservation. J Biotechnol.

[11] Davies OG, Smith AJ, Cooper PR, Shelton RM, Scheven BA (2014). The effects of cryopreservation on cells isolated from adipose, bone marrow and dental pulp tissues. Cryobiology.

[12] Iwatani M, Ikegami K, Kremenska Y, Hattori N, Tanaka S, Yagi S, Shiota K (2006). Dimethyl sulfoxide has an impact on epigenetic profile in mouse embryoid body. Stem Cells.

[13] Adler S, Pellizzer C, Paparella M, Hartung T, Bremer S (2006). The effects of solvents on embryonic stem cell differentiation. Toxicol In Vitro.

[14] Thirumala S, Wu X, Gimble JM, Devireddy RV (2010). Evaluation of polyvinylpyrrolidone as a cryoprotectant for adipose tissue-derived adult stem cells. Tissue Eng Part C Methods.

[15] Liu Y, Xu X, Ma X, Martin-Rendon E, Watt S, Cui Z (2010). Cryopreservation of human bone marrow-derived mesenchymal stem cells with reduced dimethylsulfoxide and well-defined freezing solutions. Biotechnol Prog.

[16] Marquez-Curtis LA, Janowska-Wieczorek A, McGann LE, Elliott JA (2015). Mesenchymal stromal cells derived from various tissues: biological, clinical and cryopreservation aspects. Cryobiology.

[17] Hunt CJ (2011). Cryopreservation of human stem cells for clinical application: a review. Transfus Med Hemother.

[18] Antebi B, Cheng X, Harris JN, Gower LB, Chen XD, Ling J (2013). Biomimetic collagen-hydroxyapatite composite fabricated via a novel perfusion-flow mineralization technique. Tissue Eng Part C Methods.

[19] Antebi B, Rodriguez LA, Walker KP, Asher AM, Kamucheka RM, Alvarado L, Mohammadipoor A, Cancio LC (2018). Short-term physiological hypoxia potentiates the therapeutic function of mesenchymal stem cells. Stem Cell Res Ther.

[20] Antebi B, Walker KP, Mohammadipoor A, Rodriguez LA, Montgomery RK, Batchinsky AI, Cancio LC (2018). The effect of acute respiratory distress syndrome on bone marrow-derived mesenchymal stem cells. Stem Cell Res Ther.

[21] Antebi B, Zhang Z, Wang Y, Lu Z, Chen XD, Ling J (2015). Stromal-cell-derived extracellular matrix promotes the proliferation and retains the osteogenic differentiation capacity of mesenchymal stem cells on three-dimensional scaffolds. Tissue Eng Part C Methods.

[22] Galipeau J (2013). The mesenchymal stromal cells dilemma–does a negative phase III trial of random donor mesenchymal stromal cells in steroid-resistant graft-versus-host disease represent a death knell or a bump in the road?. Cytotherapy.

[23] Francois M, Copland IB, Yuan S, Romieu-Mourez R, Waller EK, Galipeau J (2012). Cryopreserved mesenchymal stromal cells display impaired immunosuppressive properties as a result of heat-shock response and impaired interferon-gamma licensing. Cytotherapy.

[24] Quimby JM, Webb TL, Habenicht LM, Dow SW (2013). Safety and efficacy of intravenous infusion of allogeneic cryopreserved mesenchymal stem cells for treatment of chronic kidney disease in cats: results of three sequential pilot studies. Stem Cell Res Ther.

[25] Chinnadurai R, Garcia MA, Sakurai Y, Lam WA, Kirk AD, Galipeau J, Copland IB (2014). Actin cytoskeletal disruption following cryopreservation alters the biodistribution of human mesenchymal stromal cells in vivo. Stem Cell Rep.

[26] Chinnadurai R, Copland IB, Garcia MA, Petersen CT, Lewis CN, Waller EK, Kirk AD, Galipeau J (2016). Cryopreserved mesenchymal stromal cells are susceptible to T-cell mediated apoptosis which is partly rescued by IFNgamma licensing. Stem Cells.

[27] Antebi B, Benov A, Mann-Salinas EA, Le TD, Cancio LC, Wenke JC, Paran H, Yitzhak A, Tarif B, Gross KR (2016). Analysis of injury patterns and roles of care in US and Israel militaries during recent conflicts: two are better than one. J Trauma Acute Care Surg.

[28] Ginis I, Grinblat B, Shirvan MH (2012). Evaluation of bone marrow-derived mesenchymal stem cells after cryopreservation and hypothermic storage in clinically safe medium. Tissue Eng Part C Methods.

[29] Sohn HS, Heo JS, Kim HS, Choi Y, Kim HO (2013). Duration of in vitro storage affects the key stem cell features of human bone marrow-derived mesenchymal stromal cells for clinical transplantation. Cytotherapy.

[30] Galvez-Martin P, Hmadcha A, Soria B, Calpena-Campmany AC, Clares-Naveros B (2014). Study of the stability of packaging and storage conditions of human mesenchymal stem cell for intra-arterial clinical application in patient with critical limb ischemia. Eur J Pharm Biopharm.

[31] Pogozhykh D, Pogozhykh O, Prokopyuk V, Kuleshova L, Goltsev A, Blasczyk R, Mueller T (2017). Influence of temperature fluctuations during cryopreservation on vital parameters, differentiation potential, and transgene expression of placental multipotent stromal cells. Stem Cell Res Ther.

[32] Killer MC, Nold P, Henkenius K, Fritz L, Riedlinger T, Barckhausen C, Frech M, Hackstein H, Neubauer A, Brendel C (2017). Immunosuppressive capacity of mesenchymal stem cells correlates with metabolic activity and can be enhanced by valproic acid. Stem Cell Res Ther.

[33] Kaplan A, Sackett K, Sumstad D, Kadidlo D, McKenna DH (2017). Impact of starting material (fresh versus cryopreserved marrow) on mesenchymal stem cell culture. Transfusion.

[34] Samuelsson H, Ringden O, Lonnies H, Le Blanc K (2009). Optimizing in vitro conditions for immunomodulation and expansion of mesenchymal stromal cells. Cytotherapy.

[35] Matthay MA, Calfee CS, Zhuo H, Thompson BT, Wilson JG, Levitt JE, Rogers AJ, Gotts JE, Wiener-Kronish JP, Bajwa EK (2018). Treatment with allogeneic mesenchymal stromal cells for moderate to severe acute respiratory distress syndrome (START study): a randomised phase 2a safety trial. Lancet Respir Med.

[36] Moll G, Alm JJ, Davies LC, von Bahr L, Heldring N, Stenbeck-Funke L, Hamad OA, Hinsch R, Ignatowicz L, Locke M (2014). Do cryopreserved mesenchymal stromal cells display impaired immunomodulatory and therapeutic properties?. Stem Cells.

[37] Luetzkendorf J, Nerger K, Hering J, Moegel A, Hoffmann K, Hoefers C, Mueller-Tidow C, Mueller LP (2015). Cryopreservation does not alter main characteristics of Good Manufacturing Process-grade human multipotent mesenchymal stromal cells including immunomodulating potential and lack of malignant transformation. Cytotherapy.

[38] Barroso J, Osborne B, Teramura G, Pellham E, Fitzpatrick M, Biehl R, Yu A, Pehta J, Slichter SJ (2018). Safety evaluation of a lyophilized platelet-derived hemostatic product. Transfusion.

[39] Bissoyi A, Kumar A, Rizvanov AA, Nesmelov A, Gusev O, Patra PK, Bit A (2016). Recent advances and future direction in lyophilisation and desiccation of mesenchymal stem cells. Stem Cells Int.

[40] Mohammadipoor A, Antebi B, Batchinsky AI, Cancio LC (2018). Therapeutic potential of products derived from mesenchymal stem/stromal cells in pulmonary disease. Respir Res.

